# Pregnancy Outcome during the First COVID 19 Lockdown in Vienna, Austria

**DOI:** 10.3390/ijerph18073782

**Published:** 2021-04-05

**Authors:** Sylvia Kirchengast, Beda Hartmann

**Affiliations:** 1 Department of Evolutionary Anthropology, University of Vienna, A-1090 Vienna, Austria; 2 Danube Hospital, A-1220 Vienna, Austria; beda.hartmann@gesundheitsverbund.at

**Keywords:** COVID 19 pandemic in Austria, birth weight, preterm birth, maternal gestational weight gain

## Abstract

The COVID 19 pandemic represents a major stress factor for non-infected pregnant women. Although maternal stress during pregnancy increases the risk of preterm birth and intrauterine growth restriction, an increasing number of studies yielded no negative effects of COVID 19 lockdowns on pregnancy outcome. The present study focused on pregnancy outcome during the first COVID 19 lockdown phase in Austria. In particular, it was hypothesized that the national lockdown had no negative effects on birth weight, low birth weight rate and preterm birth rate. In a retrospective medical record-based single center study, the outcome of 669 singleton live births in Vienna Austria during the lockdown phase between March and July 2020 was compared with the pregnancy outcome of 277 live births at the same hospital during the pre-lockdown months of January and February 2020 and, in addition, with the outcome of 28,807 live births between 2005 and 2019. The rate of very low gestational age was significantly lower during the lockdown phase than during the pre-lockdown phase. The rate of low gestational age, however, was slightly higher during the lockdown phase. Mean birth weight was significantly higher during the lockdown phase; the rates of low birth weight, very low birth weight and extremely low birth weight were significantly lower during the lockdown phase. In contrast, maternal gestational weight gain was significantly higher during the lockdown phase. The stressful lockdown phase in Austria seems to have no negative affect on gestational length and newborn weight among non-infected mothers.

## 1. Introduction

We are currently confronted with first global pandemic since the “Spanish flu” in 1918. COVID 19 represents a major socio-economic-political-emotional stress factor (SEPE) [1] and, above all, a major worldwide health problem. This applies not only to those people infected with SARS COV 2 who suffer from a severe course of disease, but also to people who are exposed to massive stress due to psychosocial and economic consequences of the pandemic [2].

This was especially true of pregnant women during the first and second wave the of pandemic in 2020. Pregnant women suffered from a twofold stress situation: on the one hand, there is the awareness that COVID 19 infections may be deadly diseases, and the uncertainty as to whether the infection can be transmitted to the fetus and whether it has unknown consequences for the course of pregnancy and the birth and the development of the child. Furthermore, a vaccination was not available until January 2021 and is still not available on a large scale. On the other hand, the dramatic collapse of the world economy led to a state of emergency, triggered by fears, worries and suddenly occurring economic problems [1]. Maternal stress during pregnancy, however, creates adverse prenatal environmental patterns which may influence fetal growth and development in an adverse manner and also may result in long-term transgenerational consequences such as increasing offspring morbidity and mortality in later life [3,4,5,6,7,8].

Birthweight represents a sensitive indicator for the intrauterine growth process, and it is well documented that stress phases during pregnancy may result in low birth weight [9,10]. Every year, around 15 million children are born preterm or small for gestational age (SGA) worldwide, which means newborns who are smaller in size than normal for their gestational age [11]. A birth weight of less than 2500 g is considered low, and less than 1500 g is considered very low [12]. The lower the birth weight, the higher the immediate morbidity and mortality risk [13,14]. Adverse long-term consequences can also be expected. A low birth weight is therefore a major concern in obstetrics, neonatology, pediatrics and public health. Therefore, it may be assumed that a major stress situation such as the current COVID 19 pandemic may adversely affect intrauterine development and increase preterm birth rates as well as low birthweight rates.

In-utero exposure to the “Spanish flu”, which was seen as a quasi-experiment for studying the influence of a pandemic on fetal development, had adverse effects on intrauterine growth such as lower birthweights [15,16,17]. Some studies that focus on the effects of COVID 19 on pregnancy, intrauterine development and childbirth analyze potential effects of the infection during pregnancy on pregnant women, fetuses and newborns [18,19,20,21,22,23,24]. Although Sentilhes et al. reported a positive association between COVID 19 infections in pregnancy and maternal morbidity and preterm birth [24], according to recent studies, there are low rates of maternal and neonatal mortality and vertical transmission with SARS-CoV-2 [25]. The COVID 19 pandemic, however, is also a general stress factor, even for uninfected people. Chinese studies suggest that pregnant women experienced significantly increased stress levels, anxiety and depression during the COVID 19 outbreak [26]. These high stress levels may have adversely impacted the course of pregnancy, intrauterine development, parturition and birth weight even among healthy, uninfected pregnant women. Accordingly, the preterm birth rate might increase and intrauterine growth restriction and, in particular, low birth weight might become more frequent.

Consequently, an adverse effect of the COVID lockdowns on pregnancy outcome might be assumed. Interestingly, the opposite was found in several studies. While few studies reported increased stillbirth rates associated with COVID lockdowns in Italy [27], Israel [28] or UK [29], several other studies could not prove adverse effects of COVID lockdowns on pregnancy outcome. In contrast, the rate of extremely and moderately premature births decreased during COVID 19 lockdowns in many countries, such as in Ireland [30], Denmark [31], the Netherlands [20], Israel [32], Sweden [33] and the United States [34,35,36]. Furthermore, the prevalence of low birth weight decreased during the first lockdown periods, as presented in an Irish study [37]. In addition, studies from China [38] and Botswana [39] reported no increase in adverse perinatal outcomes during or after lockdown periods.

In the present study we focused on the situation in Austria, where a nationwide hard lockdown started in mid-March 2020, with severe effects on social life and the economic situation such as social distancing, loneliness and dramatically increasing unemployment rates. The main focus of the present study was pregnancy outcome during this first lockdown phase in Vienna, Austria. Based on the results of the studies mentioned above, we tested the following hypothesis: the first national lockdown in Austria from March 16 to June 15 had no negative effects on mean birth weights and the prevalence of low birthweight and pre-term birth among non-infected women in Vienna, Austria.

## 2. Materials and Methods

### 2.1. Study Setting—The First Lockdown in Austria

On 25 February 2020, the first two cases of COVID 19 infection were detected in Austria. Later, it emerged that infections had already taken place in Tyrol on 8 February. The Austrian government advised people in Austria to follow strict hand hygiene rules and to practice social distancing. The first death caused by COVID 19 was confirmed on 12 March in Vienna. On 16 March, the first complete lockdown began in Austria, which in its strict form lasted until 20 April. Schools and universities were closed and had to change to distance learning. Occupational activities (if possible) were carried out in home office. Shops, with the exception of groceries and pharmacies, were completely closed. Nationwide, homes were to be left only for necessary professional activities, necessary purchases (food or medication), assisting other people and short recreational activities in nature. According to the analysis of phone mobility records, the mobility of Austrian people dropped down drastically during the lockdown in March. Data from the Google Covid 19 Community Mobility Reports revealed that mobility was reduced by 80 percent compared to the months of January and February and remained low until the end of April. In May, there was an increase in mobility, but it was still below the comparison period before the pandemic [40].

Starting on 6 April, everyone entering a shop had to wear a face mask. On 14 April, wearing face masks became mandatory on public transportation. By late April, new cases had stabilized to around 20–50 per day on average. A slight relaxation of the lockdown measures began on 20 April, and phase two of the lockdown started. The lockdown in Austria was not over until 15 June; however, social distancing remained, as did the awareness that COVID 19 diseases are still not treatable and represent a deadly danger.

### 2.2. Data Sets

Data set 1 comprised all singleton life births (*n* = 945) taking place between 1 January and 31 July at the Viennese Danube Hospital. In this group, maternal age at birth ranged from 17 to 47 years (x = 30.1, SD = 5.3). A total of 669 of these births took place during the months March and July 2020 during the lockdown phase in Austria. A total of 277 births took place during the pre-lockdown months January and February 2020. The Danube Hospital is one of the largest public birth clinics in Vienna [41] and can therefore be regarded as representative for pregnancy outcome in Vienna. We focused on singleton births because preterm birth and low birth weight are often associated with multiple pregnancies. These two parameters among singleton live births are a much stronger indicator of stress factors affecting the mother during pregnancy. Therefore, the following inclusion criterion was defined: singleton live birth. On the other hand, a COVID 19 infection of the mother was a strict exclusion criterion. Consequently, none of the mothers was infected with COVID 19. Pregnant women have been subjected to COVID tests and antibody analyses. Women infected with COVID 19 gave birth in a specialized focus hospital during the study period and not at the Danube hospital. To compare pregnancy outcome during the first lockdown months with the pregnancy outcome before the COVID 19 pandemic, an additional dataset of 28,807 singleton live births taking place at the Danube Hospital in Vienna, Austria, between 1 January 2005 and 31 December 2019 was used. Maternal age at birth ranged from 17 to 46 years (x = 30.5, SD = 5.8). The study was carried out according to the Helsinki Declaration and is part of a large project approved by the bioethical committee of the City of Vienna (Projectnumber: (EK 19-274-VK). In addition, low birth weight (LBW) rates and Caesarean section (CS) rates in Austria between 2005 and 2019 provided by Statistics Austria [42] were used for comparison.

### 2.3. Obstetrical Characteristics and Newborn Parameters

The recorded obstetrical characteristics were gestational age at birth, newborn size and weight status and the mode of delivery. Gestational age was calculated as the difference between date of delivery and date of the last menstrual bleeding (i.e., duration of amenorrhoea) and by two consecutive ultrasound examinations performed before the twelfth week of gestation. Preterm birth was defined as ≤36 weeks of gestation. Moderate to late preterm birth (MlPB) was defined as 32 to 36 gestational weeks. Very preterm (VPB) was defined as 28 to 32 weeks of gestation, extremely preterm (EPB) as less than 28 weeks of gestation [43]. In the present study very preterm and extremely preterm were considered as one group (VPB) because only four births occurred during the 27th week of gestation. We also include CS. All Caesarean sections were carried out exclusively for medical reasons, such as cephalo-pelvic disproportion (diagnosed by sonography), adverse child presentation or placenta previa. Caesarean sections upon maternal request without any medical indication were not performed at the Danube Hospital [27]. The most frequent indications for emergency Caesarean delivery were fetal distress and obstructed labor.

All newborns were measured immediately after birth. The following parameters were directly taken from the newborn: birth weight in grams using a digital infant scale, birth length in centimeters using a standard measurement board for infants and head circumference in centimeters using a tape. A low birth weight (LBW) was defined as 1500–2500 g, a very low birth weight (VLBW) as 1000–1500 g, and an extremely low birth weight (ELBW) as <1000 g according to the recommendations of the World Health Organization (WHO) [11].

### 2.4. Maternal Parameters

The following maternal parameters were collected: age at giving birth, body height, pre-pregnancy weight, end of pregnancy weight and gestational weight gain. Body height was measured to the nearest 0.1 cm using a standard anthropometer. Pre-pregnancy weight was obtained by interview using the retrospective method. Body weight was measured again to the nearest 0.1 kg on a balance beam scale, at the first prenatal visit around the eighth week of gestation. Additionally, maternal weight was measured before delivery (i.e., at the end of pregnancy). The weight gain during pregnancy was calculated by subtracting pre-pregnancy weight from body weight before delivery. Pre-pregnancy body mass index (PPBMI) was calculated ((body weight in kg)/(body height in m)^2^).

### 2.5. Socio-Economic Indicators

Since no personal socioeconomic data of the mothers, such as employment, income or educational level, were available, the unemployment rate in Austria, published by Statistics Austria [42], was used as an indicator of the general economic situation during the lockdown months in Austria.

### 2.6. Study Design

The study design corresponds to a retrospective medical record-based single-centre study. In a first step, pregnancy outcome (newborn size, gestational age at birth), Caesarean section rate and maternal somatic parameters (age, body height, body weight, pre-pregnancy body mass index and gestational weight gain) at the Danube hospital during the lockdown months March to July 2020 were compared to pregnancy outcome during the pre-lockdown months January and February 2020. Furthermore, pregnancy outcome during the lockdown months was compared with pregnancy outcome between 2005 and 2019 at the Danube hospital in order to provide a long-term perspective of the pregnancy outcome at this medical center. In addition, low birthweight rate and Cesarean section rate at the Danube hospital between 2005 and 2019 were compared with low birthweight rate and Caesarean section rate in Austria using data from Statistics Austria. In a second step, association patterns between pregnancy outcome and maternal parameters as well as lockdown phase were tested. Furthermore, the unemployment rate during the lockdown phase is presented.

### 2.7. Statistical Analyses

Statistical analyses were carried out using SPSS for Windows (version 26.00, IBM, Vienna, Austria). The Kolmogorov–Smirnov test indicated the normal distribution of most metric variables. Therefore, parametric tests were performed exclusively. After computing descriptive statistics, Student’s *t*-tests and χ^2^ were calculated to test differences between the births during the lockdown phase and those during pre-lockdown period. Additionally, linear regression analyses were calculated to analyze the associations between maternal parameters as well as the lockdown and gestational age at birth and newborn weight. *p* < 0.05 was considered statistically significant.

## 3. Results

### 3.1. Gestational Age

Table 1 shows the rates of late or moderate preterm births (MLPB) (32 to 36 weeks) and very preterm birth (VPB) (<32 weeks) of the last 15 years (2005–2019) and pre-lockdown as well as the lockdown months of 2020. The highest rate of VPB was found in 2015 with 30.1/1000, and the lowest rate (7.9/1000) in 2005. In January and February 2020, before the Austrian lockdown, the VPB rate was quite high with 28.9/1000. During the lockdown, however the rate of VPB dropped to 14.9/1000. Comparing the pre-lockdown months and the lockdown months of 2020, the risk of VPB was markedly higher during the pre-lockdown months (OR 1.92, CI 0.76–4.79). The lowest rate of MLPB (47.6/1000) was found in 2009, the highest in 2017 (77.2/1000). During the pre-lockdown months January and February, the MLPB rate was quite low (54.2/1000) and increased during the lockdown months of March to July to 63/1000. The risk of MLPB, however, was only slightly higher during the lockdown months (OR 1.01, CI 0.97–1.05). Sixty-three per thousand, however, was not the highest MLPB rate during the last 15 years.

Examining the individual months of 2020, the VPB rate was lower during the lockdown months of March to June than during January/February. The MLBP rate, in contrast, was highest during the lockdown months of May and June 2020, but dropped during July 2020 (Table 2).

### 3.2. Birthweight

**Figure 1:**
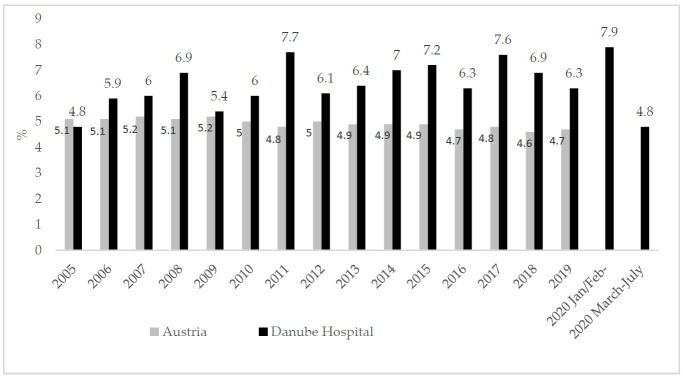
Prevalence of birth weight below 2500 g in Austria (data source: [42]) and in the Danube Hospital 2005 to 2020.

According to the Statistics Austria dataset, the prevalence of low birthweight among singleton births in Austria ranged between 4.7 and 5.2% from 2005 to 2019. The prevalence of low birth weight at the Danube Hospital during this time span was higher, ranging between 4.8% in 2005 and 7.9% in January/February 2020 (Figure 1).

Comparing the rate/1000 LBW between the pre-lockdown months of January/February 2020 and the lockdown months of March to July 2020 showed a significantly higher rate (*p* = 0.049) of LBW in January/February (54.2/1000) than during the lockdown period of March to July (38.9/1000). The risk of giving birth to a LBW newborn was significantly higher during pre-lockdown period (OR 1.66 CI 0.98–2.81). The rates of extremely low birthweight (<1000 g), very low birthweight (1000–1500 g) and low birthweight (1501–2500 g) at the Danube hospital are listed in Table 1. The comparison of the pre-lockdown months of January/February 2020 and the lockdown months of March to July 2020, yielded markedly higher risks of ELBW (OR 2.48 CI 0.35–17.50), VLBW (OR 2.05 CI 0.63–6.67) and LBW (OR 1.41 CI 0.76–2.62) during the pre-lockdown months. The rates of low, very low and extremely low birthweight between January and July 2020 are listed in Table 2.

### 3.3. Newborn Size

The mean birth weight at the Danube Hospital was 3344 g during the last 15 years. The lowest mean births weight (3322.2 g) occurred in 2017. The highest mean birth weight occurred during the lockdown phase (3381.7 g). A similar trend was evident for birth length, with the highest values (50.8 cm) also reported for the lockdown period. Mean head circumference (34.3 cm) did not differ markedly between the years 2005 to 2020 (Table 3).

The comparison of newborn size between pre-lockdown and lockdown months in 2020 yielded insignificantly higher birthweight and insignificantly higher birth lengths during the lockdown months. Newborn head circumference was only slightly higher during the lockdown months.

### 3.4. Caesarean Section Rates

**Figure 2:**
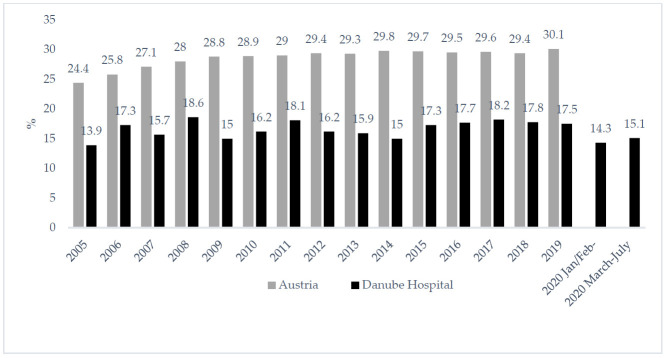
Prevalence of Caesarean section rate in Austria [42] and in the Danube Hospital 2005 to 2020.

During the last fifteen years, the Caesarean section rates at the Danube hospital were always markedly lower than the Caesarean section rate of Austria as a whole. (Figure 2). During the lockdown months, the Caesarean section rate did not increase markedly at the Danube hospital. In fact, the low CS rate at that hospital (compared to overall Austria) remained low during the lockdown months between March and July 2020; Caesarean sections were performed only in 14.1% of the births.

### 3.5. Maternal Somatic Parameters

Age, body height, body weight and pre-pregnancy BMI did not differ significantly between mothers giving births during the pre-lockdown months January/February 2020 and mothers giving birth during the lockdown months. Gestational weight gain, however, differed significantly (Table 4). During the lockdown months, the mean gestational weight gain was 14.2 kg, i.e., an increase of 1 kg compared with January/February 2020. This difference in weight gain between the pre-lockdown and lockdown months was statistically significant (*p* = 0.021).

In general, 14.2 kg was the highest gestational weight gain during the last 15 years (Table 5). Furthermore, the percentage of women who experienced a gain of more than 15 kg during pregnancy was significantly higher during the lockdown months (*p* = 0.049). Nearly 39% of the pregnant women showed a weight gain of more than 15 kg between March and July 2020, while this was true of only 33.8% of the mothers during the pre-lockdown months (Table 5).

### 3.6. Associations between Pregnancy Outcome and Maternal Parameters

Linear multiple regression analyses yielded that birth weight in 2020 was significantly positively associated with maternal gestational weight gain (Coefficient B = 11.66, *p* = 0.001, 95% CI 7.43 to 15.89), but not with maternal age and the lockdown phase. The same associations could be observed for gestational length. Gestational length was significantly positively associated with maternal gestational weight gain (Coefficient B = 0.05; *p* = 0.001; 95% CI = 0.03 to 0.07), while no significant association with the maternal age and the lockdown phase could be observed. Consequently, gestational weight gain seems to be a key factor for pregnancy outcome in this sample.

## 4. Discussion

After the first case of coronavirus infection was detected in Wuhan, China, in December 2019, the disease spread all over the world within a few weeks. The WHO declared Coronavirus Infection Disease 2019 (COVID 19) a Public Health Emergency of international concern on 30 January 2020 and, subsequently, a pandemic on 11 March 2020. COVID 19 not only represents a pandemic and global health crisis but is also a psychosocial and economic disaster [1]. Stress, worries and anxieties during pregnancy are often associated with intrauterine growth restriction and/or preterm birth [44,45,46,47]. Economic crises, such as the financial crisis of 2008, also led to reduced mean birth weights in particularly affected countries such as Spain [48,49,50], Greece [51], Portugal [52], Iceland [53], Japan [54], Argentina [55], Brazil [56] and the USA [57]. Since the economic effects of the COVID 19 pandemic are comparable to those of the financial crisis in 2008, the COVID 19 pandemic and, in particular, the lockdown phases probably result in similar effects on intrauterine growth and pregnancy outcome. Interestingly, several studies yielded no negative effects of the COVID 19 lockdowns on pregnancy outcome [27,28,29,30,31,32,33,34,35,36,37,38,39].

In the present study, pregnancy outcome during the first hard lockdown phase in Austria was focused on. In particular, the following hypothesis was tested: the first national lockdown in Austria from March 16 to June 15 had no negative effects on mean birth weights and the prevalence of low birthweight and pre-term birth among non-infected women in Vienna, Austria. Exclusively non-infected mothers were examined in order to exclude effects of COVID 19 infections on the fetus. The comparison of births during the lockdown months March to July with the pre-lockdown situation (January and February 2020) and the database from the last 15 years supported the unexpected results of previous studies [27,28,29,30,31,32,33,34,35,36,37,38,39]. The rate of preterm birth did not increase significantly during the lockdown; in contrast, the rate of VPB during the lockdown months was markedly lower than in the years before. These findings are in accordance with previous studies from Israel [32], Denmark [31], UK [29] and the United states [35] that also reported either no change or a decrease in extremely low gestational age. For Italy, a significant decrease in late preterm births (23 to 36 gestational week) was reported [27]. In the present study, no significant decrease in late preterm birth could be observed. Late preterm birth rates during the lockdown were slightly lower than between 2016 and 2019 but higher than during the pre-lockdown months of January and February 2020.

Mean weight and length at birth were highest during the lockdown months, whereas the rates of low birthweight (1500 to 2500 g), very low birthweight (1000 to 1500 g) and extremely low birth weight (<1000 g) decreased during those months. These findings are in contrast to the observations during the financial crisis 2008 [47,48,49,50,51,52,53], but in accordance with the results of Philip et al. [37]. In that Irish study, an unpredicted 73% reduction in live births of VLBW newborns and a 100% reduction of ELBW newborns during the Irish lockdown was observed. In addition, the Caesarean section rates remained low in the Austrian sample during the lockdown, indicating no increase in complications during deliveries.

Although the results of the present study are in accordance with those of some previous studies [27,28,29,30,31,32,33,34,35,36,37,38,39], the lack of negative effects of the lockdown phase on pregnancy outcome is rather unexpected. The COVID 19 pandemic produced social and economic stress; both are particular risk factors for preterm birth and giving birth to growth-restricted newborns [58]. The impact of global pandemics on fetal growth and a transgenerational effect were described for the influenza pandemic of 1918. People born in 1919 who were exposed to the influenza pandemic in utero expressed low birth weight and also worse health and higher mortality in older age [15,58,59,60]. Although COVID 19 is the first global pandemic since 1918, the situation in Austria in 2020 is quite different. Both the medical care and the affords taken by the government to cushion the economic consequences of the pandemic cannot be compared with the situation in 1918. Nonetheless, unemployment rates increased dramatically within few days in March 2020, whereas in February 333,987 people were unemployed in Austria, that number rose to 504,345 in March [42]. The highest value was reported in April (522,253). In May, the unemployment rate decreased with the end of phase one of the lockdown. The COVID 19 pandemic did not only create economic stress, people were also confronted with a new, unknown and deadly virus infection. Although during the lockdown months of March to July the primary focus has been on typical vulnerable groups such as elderly and patients with underlying medical conditions, an infection during pregnancy was seen as a risk for the pregnant women and the fetus [18]. The effects of a possible maternal–fetal transmission of SARSCOV 2 infections were still unclear [22]. Some studies suggested an association between a SARSCOV 2 infection during pregnancy and maternal morbidity and preterm birth [20,24], but also adverse birth outcomes and increased caesarean section rates [22]. The fear of a new deadly disease and economic problems caused by an unexpected increase in the unemployment rate due to COVID 19 created stress, which may have negative consequences for pregnancy outcome [61,62,63,64,65].

In the present study, the lockdown related stress could have affected the fetuses mainly during the third trimester of pregnancy. Previous studies suggested that maternal and intrauterine environments affect fetal growth in weight mainly during the third trimester [66]. This is mainly due to compromised uteroplacental perfusion and the exposure to high maternal cortisol levels. Therefore, increased maternal stress during late pregnancy should be associated with increased rates of preterm birth and low birth weight. Interestingly, the lockdown showed no negative effects on gestational age and birthweight. In contrast, preterm birth rates and low birthweight rates decreased during the lockdown phases not only in Austria, but also in several other countries [28,29,30,31,32,33,34,35,36,37,38,39]. The reasons for the decrease in preterm birth rates and the increase in newborn size during the COVID 19 lockdowns are still unclear. Although we cannot support our interpretation with data, we suggest that the COVID 19 lockdown forced behavioral changes and life style modifications in pregnant women. The study design did not allow testing for associations or causality between life style changes and the unexpected increase in birth weight and gestational length. Berghella et al. [35] postulated several hypotheses, such as reduced work hours, reduced somatic and emotional stress of work, increased support by the family, reduced load of infections, better nutrition or governmental financial support. The same issue was relevant in Philip et al. [37]. Importantly, we do have one hint of changes in the maternal factors: gestational weight gain increased significantly during the lockdown. Although mothers giving birth during the pre-lockdown months of January and February 2020 and mothers giving birth during the lockdown months of March to July 2021 did not differ significantly in age, body height and pre-pregnancy body mass index, mothers during the lockdown phase gained significantly more weight. On the one hand this may be due to longer gestational length, but we suppose that gestational weight gain may be used as a proxy for behavioral changes. Social distancing and the home office situation affected the behavior of pregnant women. Pregnant women have been forced to stay at home, potentially decreasing physical activity and increasing their nutritional intake, which might result in increased gestational weight gain. This effect was described by Zhang et al., who reported an increase in emotional eating among pregnant women during the COVID 19 pandemic. Decreases in physical activity but increased worries about the pandemic caused emotional eating behavior, which was associated with increased gestational weight gain [67]. Stressful situations are often associated with changes in diet and eating behavior. The fear of COVID 19 and increased depression rates were positively associated with emotional eating, characterized by an intake of high sugar foods and beverages [68,69]. In the present study, we have no information concerning depression and food intake among pregnant women, but we clearly see an increased gestational weight gain during the COVID 19 lockdown. This increased gestational weight gain seems to be a key factor for pregnancy outcome during the lockdown months. The regression analyses clearly showed that the gestational weight gain was significantly associated with birth weight and gestational length, while the lockdown phase was not significantly related to birthweight and gestational length. On the other hand, financial support from the Austrian government helped to reduce the immediate economic consequences. Bogin and Varea [1] declared COVID 19 to be an SEPE. In our opinion, this first lockdown stress to pregnant women may have been lower than expected in Austria.

Our study contains the following limitations. A main issue is the retrospective design, which does not enable including standardized stress inventories or questionnaires to obtain information concerning economic parameters and the individual perception of the lockdown by the pregnant women. Furthermore, we have no information regarding the family situation, the perceived social support or the cultural and ethnic background of the mothers. The study design did not allow testing causalities or even associations between stress factors, behavior life style and pregnancy outcome of the study population. The focus group—deliveries during the lockdown months—was relatively small. Only 669 singleton births took place during the lockdown phase at the Danube Hospital. Although the hospital is one of the largest public birth clinics in Vienna, the study population is not representative for whole Austria, and the findings should not be generalized. Nevertheless, the study also has strengths. This is the first study analyzing pregnancy outcome among uninfected women during the first hard COVID 19 lockdown in Austria. This gives it a pilot study character, and its design made a comparison with the rates of low birth weight and preterm births over the last 15 years.

## 5. Conclusions

Neither the extremely preterm births rates nor the prevalence of low birth weight increased during the lockdown months. On the contrary, both parameters were lower during the lockdown months than in the years before. Gestational weight gain, however, was significantly higher during the lockdown months. Furthermore, gestational weight gain was significantly associated with birthweight and gestational length. The stressful lockdown phase in Austria seems to have no significantly negative effect on extremely preterm birth rates and newborn weight among non-infected mothers.

## Figures and Tables

**Table 1:** Rates of ELBW (<1000 g), VLBW (<1500 g), LBW (1500–2500 g), VPB (<32 weeks), MLPB (32–36 week) at the Danube Hospital between 2005 and 2019.

Year	Singleton Live Births *n*	ELBW		VLBW		LBW		VPB		MLPB	
		*n*	Rate/1000	*n*	Rate/1000	*n*	Rate/1000	*n*	Rate/1000	*n*	Rate/1000
2005	1507	2	1.3	8	5.3	64	42.5	12	7.9	87	57.7
2006	1767	8	4.5	13	7.4	87	49.2	30	16.9	116	65.6
2007	1811	8	4.4	21	11.6	82	45.3	40	22.1	104	57.4
2008	1848	10	5.4	19	10.3	100	54.1	45	24.4	107	57.9
2009	1850	8	4.3	13	7.0	85	45.9	36	19.5	88	47.6
2010	1805	7	3.9	14	7.8	90	49.9	34	18.8	98	54.3
2011	1917	9	4.7	30	15.6	108	56.3	49	25.6	122	63.6
2012	1927	6	3.1	16	8.3	104	53.9	40	20.8	118	61.2
2013	2015	10	4.9	14	6.9	110	54.6	44	21.8	107	53.1
2014	2045	11	5.4	21	10.3	118	57.7	52	25.4	110	53.8
2015	2185	11	5.0	28	12.8	122	55.8	66	30.2	130	59.5
2016	2232	7	3.1	24	10.7	117	52.4	44	19.7	145	64.9
2017	2164	18	8.3	22	10.2	125	57.8	57	26.3	167	77.2
2018	2186	9	4.1	12	5.5	132	60.4	38	17.4	140	64.0
2019	1547	4	2.6	14	9.0	80	51.7	28	18.1	98	63.3
2020 Jan/Feb	277	2	7.2	5	18.0	15	54.2	8	28.9	15	54.2
2020 March–July	669	2	2.9	6	8.9	26	38.9	10	14.9	42	62.9

Legend: ELBW = extremely low birth weight (<1000 g), VLBW = very low birth weight (1000–1499 g), LBW = low birthweight (1500–2500 g), VPB= very preterm birth (<32 weeks), MLPB = moderate or late preterm birth (32–36 weeks).

**Table 2:** Rates of ELBW (<1000 g), VLBW (<1500 g), LBW (1500–2500 g), VPB (<32 weeks) MLPB (32–36 week) and CS at the Danube Hospital between January and July 2020.

Month	Singleton Live Births *n*	ELBW		VLBW		LBW		VPB		MLPB		CS	
		*n*	Rate/1000	*n*	Rate/1000	*n*	Rate/1000	*n*	Rate/1000	*n*	Rate/1000	*n*	%
January	149	1	6.7	1	6.7	9	60.4	3	20.1	10	67.1	16	10.7
February	128	1	7.8	4	31.3	6	46.9	5	39.1	5	39.1	18	14.1
March	45	0	0.0	0	0.0	3	66.7	0	0	3	66.7	4	8.9
April	128	1	7.8	1	7.8	2	15.6	2	15.6	7	54.7	18	14.1
May	159	0	0.0	4	25.2	5	31.4	3	18.9	11	69.2	23	14.5
June	191	0	0.0	1	5.2	9	47.1	3	15.7	14	73.3	29	15.2
July	145	1	6.9	0	0.0	5	34.4	1	6.9	7	48.3	20	13.7

Legend: ELBW = extremely low birth weight (<1000 g), VLBW = very low birth weight (1000–1499 g), LBW = low birthweight (1500–2500 g), VPB= very preterm birth (<32 weeks), MLPB = moderate or late preterm birth (32–36 weeks), CS = Caesarean section.

**Table 3:** Newborn parameters birthweight, birth length and head circumference 2005 to 2020 descriptive statistics.

Year	*n*	Birth Weight (g)		Birth Length (cm)		Head Circumference (cm)	
		Mean	SD	Mean	SD	Mean	SD
2005	1507	3353.4	517.4	50.7	2.7	34.2	1.6
2006	1767	3350.4	554.0	50.6	2.7	34.3	1.6
2007	1811	3337.0	582.2	50.3	2.9	34.2	1.8
2008	1848	3328.1	598.8	50.3	2.7	34.2	1.8
2009	1850	3345.1	551.8	50.4	2.7	34.3	1.6
2010	1805	3333.1	557.1	50.3	2.6	34.2	1.7
2011	1917	3324.6	603.3	50.3	2.9	34.2	1.8
2012	1927	3354.0	573.9	50.4	2.9	34.1	1.8
2013	2015	3369.3	586.9	50.5	2.9	34.1	1.7
2014	2045	3345.5	608.4	50.4	3.0	34.0	1.9
2015	2185	3338.1	604.9	50.5	3.1	34.1	1.9
2016	2232	3351.2	583.7	50.5	2.9	34.1	1.9
2017	2164	3322.2	616.3	50.2	3.2	34.1	1.9
2018	2186	3354.9	577.3	50.6	2.8	34.1	1.8
2019	1547	3356.9	580.2	50.7	2.9	34.2	1.8
2020 Jan/Feb	277	3342.2	630.6	50.5	3.2	34.2	2.1
March–July	669	3381.7	556.4	50.8	2.9	34.3	2.1

**Table 4:** Maternal somatic parameters. A comparison between pre-lockdown and lockdown months in 2020 (Student’s *t*-tests).

Maternal Parameter	January/February Pre-Lockdown		March to July Lockdown		Sign.
	Mean	SD	Mean	SD	*p*-Value
Age (yrs.)	31.1	5.3	30.8	5.4	0.143
Body height (cm)	165.1	6.9	164.7	6.1	0.301
Body weight before pregnancy (kg)	67.3	15.7	66.2	14.4	0.275
Body weight end of pregnancy (kg)	80.4	15.5	80.5	14.9	0.916
Pre-pregnancy BMI (kg/m^2^)	24.69	5.59	24.41	4.98	0.433
Gestational weight gain (kg)	13.2	5.9	14.2	6.7	0.021

**Table 5:** Maternal gestational weight gain categories LGWG (<10 kg), AGWG (10–15 kg), HGWG (>15 kg) at the Danube Hospital between 2005 and 2019.

Year	Gestational Weight Gain (kg)		LGWG <10 kg	AGWG 10–15 kg	HGWG >15 kg
	x	SD			
2005	12.5	5.9	26.0%	45.3%	28.7%
2006	13.4	5.9	20.0%	48.8%	31.2%
2007	13.5	5.4	21.6%	45.8%	31.6%
2008	13.2	6.2	22.1%	47.7%	30.1%
2009	13.0	5.6	23.6%	46.8%	29.6%
2010	13.8	5.6	20.0%	45.2%	34.8%
2011	13.7	5.6	20.4%	44.9%	34.7%
2012	13.5	5.6	21.5%	45.3%	33.2%
2013	14.0	5.9	18.9%	45.1%	36.0%
2014	14.1	6.2	20.1%	45.4%	34.5%
2015	13.8	6.3	21.7%	40.8%	33.5%
2016	13.9	6.3	20.2%	47.6%	32.2%
2017	13.9	5.9	20.7%	45.7%	33.6%
2018	13.9	6.3	22.1%	45.3%	32.6%
2019	13.5	5.8	21.6%	44.8%	33.5%
2020 Jan/Feb	13.2	5.9	24.9%	41.3%	33.8%
March-July	14.2	6.7	20.3%	40.8%	38.8%

Legend: LGWG = low gestational weight gain, AGWG =average gestational weight gain, HGWG = high gestational weight gain.

## Data Availability

Restrictions apply to the availability of the data used in this study- Data was obtained from medical records of the SMZOst and are exclusively with the permission of the Wiener Gesundheitsverbund.

## References

[1] Bogin B., Varea C. (2020). COVID-19, crisis, and emotional stress: A biocultural perspective of their impact on growth and development for the next generation. Am. J. Hum. Biol..

[2] Leonard W.R. (2020). Human Biologists confront the COVID-19 pandemic. Am. J. Hum. Biol..

[3] Barker D.J.P. (1995). Fetal origins of coronary heart disease. BMJ.

[4] Hales C.N., Barker D.J.P. (2001). The thrifty phenotype hypothesis. Br. Med. Bull..

[5] Okano L., Ji Y., Riley A.W., Wang X. (2019). Maternal psychosocial stress and children’s ADHD diagnosis: A prospective birth cohort study. J. Psychosom. Obstet. Gynecol..

[6] Manzari N., Matvienko-Sikar K., Baldoni F., O’Keeffe G.W., Khashan A.S. (2019). Prenatal maternal stress and risk of neurodevelopmental disorders in the offspring: A systematic review and meta-analysis. Soc. Psychiatry Psychiatr. Epidemiol..

[7] Kamai E.M., McElrath T.F., Ferguson K.K. (2019). Fetal growth in environmental epidemiology: Mechanisms, limitations, and a review of associations with biomarkers of non-persistent chemical exposures during pregnancy. Environ. Health.

[8] Workalemahu T., Grantz K.L., Grewal J., Zhang C., Louis G.M.B., Tekola-Ayele F. (2018). Genetic and Environmental Influences on Fetal Growth Vary during Sensitive Periods in Pregnancy. Sci. Rep..

[9] Lima S.A.M., El Dib R.P., Rodrigues M.R.K., Ferraz G.A.R., Molina A.C., Neto C.A.P., De Lima M.A.F., Rudge M.V.C. (2018). Is the risk of low birth weight or preterm labor greater when maternal stress is experienced during pregnancy? A systematic review and meta-analysis of cohort studies. PLoS ONE.

[10] Rondó P.H.C., Ferreira R.F., Nogueira F., Ribeiro M.C.N., Lobert H., Artes R. (2003). Maternal psychological stress and distress as predictors of low birth weight, prematurity and intrauterine growth retardation. Eur. J. Clin. Nutr..

[11] De Onis M., Habicht J.P. (1996). Anthropometric reference data for international use: Recommendations from a World Health Organization Expert Committee. Am. J. Clin. Nutr..

[12] WHO (2004). International Statistical Classification of Diseases and Related Health Problems, Tenth Revision.

[13] Mayor S. (2016). Low birth weight is associated with increased deaths in infancy and adolescence, shows study. BMJ.

[14] McIntire D.D., Bloom S.L., Casey B.M., Leveno K.J. (1999). Birth Weight in Relation to Morbidity and Mortality among Newborn Infants. N. Engl. J. Med..

[15] Mazumder B., Almond D., Park K., Crimmins E.M., Finch C.E. (2009). Lingering prenatal effects of the 1918 influenza pandemic on cardiovascular disease. J. Dev. Orig. Health Dis..

[16] Helgertz J., Bengtsson T. (2019). The Long-Lasting Influenza: The Impact of Fetal Stress During the 1918 Influenza Pandemic on Socioeconomic Attainment and Health in Sweden, 1968–2012. Demography.

[17] Chandra S., Christensen J., Mamelund S.-E., Paneth N. (2018). Short-Term Birth Sequelae of the 1918–1920 Influenza Pandemic in the United States: State-Level Analysis. Am. J. Epidemiol..

[18] Dang D., Wang L., Zhang C., Li Z., Wu H. (2020). Potential effects of SARS-CoV -2 infection during pregnancy on fetuses and newborns are worthy of attention. J. Obstet. Gynaecol. Res..

[19] Duran P., Berman S., Niermeyer S., Jaenisch T., Forster T., De Leon R.G.P., De Mucio B., Serruya S. (2020). COVID-19 and newborn health: Systematic review. Revista Panamericana de Salud Pública.

[20] Been J.V., Ochoa L.B., Bertens L.C.M., Schoenmakers S., Steegers E.A.P., Reiss I.K.M. (2020). Impact of COVID-19 mitigation measures on the incidence of preterm birth: A national quasi-experimental study. Lancet Public Health.

[21] De Melo G.C., De Araújo K.C.G.M. (2020). COVID-19 infection in pregnant women, preterm delivery, birth weight, and vertical transmission: A systematic review and meta-analysis. Cad. Saúde Pública.

[22] Smith V., Seo D., Warty R., Payne O., Salih M., Chin K.L., Ofori-Asenso R., Krishnan S., Costa F.D.S., Vollenhoven B. (2020). Maternal and neonatal outcomes associated with COVID-19 infection: A systematic review. PLoS ONE.

[23] Yang R., Mei H., Zheng T., Fu Q., Zhang Y., Buka S., Yao X., Tang Z., Zhang X., Qiu L. (2020). Pregnant women with COVID-19 and risk of adverse birth outcomes and maternal-fetal vertical transmission: A population-based cohort study in Wuhan, China. BMC Med..

[24] Sentilhes L., De Marcillac F., Jouffrieau C., Kuhn P., Thuet V., Hansmann Y., Ruch Y., Fafi-Kremer S., Deruelle P. (2020). Coronavirus disease 2019 in pregnancy was associated with maternal morbidity and preterm birth. Am. J. Obstet. Gynecol..

[25] Huntley B.J.F., Huntley E.S., Di Mascio D., Chen T., Berghella V., Chauhan S.P. (2020). Rates of Maternal and Perinatal Mortality and Vertical Transmission in Pregnancies Complicated by Severe Acute Respiratory Syndrome Coronavirus 2 (SARS-Co-V-2) Infection. Obstet. Gynecol..

[26] Wu Y., Zhang C., Liu H., Duan C., Li C., Fan J., Li H., Chen L., Xu H., Li X. (2020). Perinatal depressive and anxiety symptoms of pregnant women during the coronavirus disease 2019 outbreak in China. Am. J. Obstet. Gynecol..

[27] De Curtis M., Villani L., Polo A. (2021). Increase of stillbirth and decrease of late preterm infants during the COVID-19 pandemic lockdown. Arch. Dis. Child. Fetal Neonatal Ed..

[28] Mor M., Kugler N., Jauniaux E., Betser M., Wiener Y., Cuckle H., Maymon R. (2021). Impact of the COVID-19 Pandemic on Excess Perinatal Mortality and Morbidity in Israel. Am. J. Perinatol..

[29] Khalil A., Von Dadelszen P., Draycott T., Ugwumadu A., O’Brien P., Magee L. (2020). Change in the Incidence of Stillbirth and Preterm Delivery During the COVID-19 Pandemic. JAMA.

[30] McDonnell S., McNamee E., Lindow S.W., O’Connell M.P. (2020). The impact of the Covid-19 pandemic on maternity services: A review of maternal and neonatal outcomes before, during and after the pandemic. Eur. J. Obstet. Gynecol. Reprod. Biol..

[31] Hedermann G., Hedley P.L., Bækvad-Hansen M., Hjalgrim H., Rostgaard K., Poorisrisak P., Breindahl M., Melbye M., Hougaard D.M., Christiansen M. (2021). Danish premature birth rates during the COVID-19 lockdown. Arch. Dis. Child. Fetal Neonatal Ed..

[32] Meyer R., Bart Y., Tsur A., Yinon Y., Friedrich L., Maixner N., Levin G. (2020). A marked decrease in preterm deliveries during the coronavirus disease 2019 pandemic. Am. J. Obstet. Gynecol..

[33] Pasternak B., Neovius M., Söderling J., Ahlberg M., Norman M., Ludvigsson J.F., Stephansson O. (2021). Preterm Birth and Stillbirth During the COVID-19 Pandemic in Sweden: A Nationwide Cohort Study. Ann. Intern. Med..

[34] Main E.K., Chang S.C., Carpenter A.M., Wise P.H., Stevenson D.K., Shaw G.M., Gould J.B. (2021). Singleton preterm birth rates for racial and ethnic groups during the coronavirus disease 2019 pandemic in California. Am. J. Obstet. Gynecol..

[35] Berghella V., Boelig R., Roman A., Burd J., Anderson K. (2020). Decreased incidence of preterm birth during coronavirus disease 2019 pandemic. Am. J. Obstet. Gynecol..

[36] Handley S.C., Mullin A.M., Elovitz M.A., Gerson K.D., Montoya-Williams D., Lorch S.A., Burris H.H. (2021). Changes in Preterm Birth Phenotypes and Stillbirth at 2 Philadelphia Hospitals During the SARS-CoV-2 Pandemic, March-June 2020. JAMA.

[37] Philip R.K., Purtill H., Reidy E., Daly M., Imcha M., McGrath D., O’Connell N.H., Dunne C.P. (2020). Unprecedented reduction in births of very low birthweight (VLBW) and extremely low birthweight (ELBW) infants during the COVID-19 lockdown in Ireland: A ‘natural experiment’ allowing analysis of data from the prior two decades. BMJ Glob. Health.

[38] Li M., Yin H., Jin Z., Zhang H., Leng B., Luo Y., Zhao Y. (2020). Impact of Wuhan lockdown on the indications of cesarean delivery and newborn weights during the epidemic period of COVID-19. PLoS ONE.

[39] Caniglia E.C., Magosi L.E., Zash R., Diseko M., Mayondi G., Mabuta J., Powis K., Dryden-Peterson S., Mosepele M., Luckett R. (2020). Modest reduction in adverse birth outcomes following the COVID-19 lockdown. Am. J. Obstet. Gynecol..

[40] Google Covid-19 Community Mobility Reports. https://www.google.com/covid19/mobility/.

[41] Kirchengast S., Hartmann B. (2018). Recent Lifestyle Parameters Are Associated with Increasing Caesarean Section Rates among Singleton Term Births in Austria. Int. J. Environ. Res. Public Health.

[42] Statistics Austria (2020). Statistical Yearbook.

[43] World Health Organization (2018). Preterm Birth. https://www.who.int/news-room/fact-sheets/detail/pretermbirth.

[44] Keasley J., Blickwedel J., Quenby S. (2017). Adverse effects of exposure to armed conflict on pregnancy: A systematic review. BMJ Glob. Health.

[45] Comacho A. (2008). Stress and Birth Weight: Evidence from Terrorist Attacks. Am. Econ. Rev..

[46] Hobel C.J., Goldstein A., Barrett E.S. (2008). Psychosocial Stress and Pregnancy Outcome. Clin. Obstet. Gynecol..

[47] Roseboom T.J., van der Meulen J.H., Ravelli A.C., Osmond C., Barker D.J., Bleker O.P. (2001). Effects of prenatal exposure to the Dutch famine on adult disease in later life: An overview. Mol. Cell. Endocrinol..

[48] Teran J.M., Varea C., Juarez S., Bernis C., Bogin B. (2018). Social disparities in low birth weight among Spanish mothers during the economic crisis (2007–2015). Nutr. Hosp..

[49] Terán J.M., Juárez S., Bernis C., Bogin B., Varea C. (2020). Low birthweight prevalence among Spanish women during the economic crisis: Differences by parity. Ann. Hum. Biol..

[50] Varea C., Terán J.M., Bernis C., Bogin B., González-González A. (2016). Is the economic crisis affecting birth outcome in Spain? Evaluation of temporal trend in underweight at birth (2003–2012). Ann. Hum. Biol..

[51] Zografaki I., Papamichail D., Panagiotopoulos T. (2018). Adverse effect of the financial crisis in Greece on perinatal factors. Eur. J. Public Health.

[52] Kana M.A., Correia S., Peleteiro B., Severo M., Barros H. (2017). Impact of the global financial crisis on low birth weight in Portugal: A time-trend analysis. BMJ Glob. Health.

[53] Olafsson A. (2016). Household Financial Distress and Initial Endowments: Evidence from the 2008 Financial Crisis. Health Econ..

[54] Ueda P., Kondo N., Fujiwara T. (2015). The global economic crisis, household income and pre-adolescent overweight and underweight: A nationwide birth cohort study in Japan. Int. J. Obes..

[55] Ratowiecki J., Poletta F.A., Giménez L.G., Gili J.A., Pawluk M.S., López Camelo J.S. (2018). Prevalence of low birth weight in a scenario of economic depression in Argentina. Arch. Argent. Pediatr..

[56] Mrejen M., Machado D.C. (2019). In utero exposure to economic fluctuations and birth outcomes: An analysis of the relevance of the local unemployment rate in Brazilian state capitals. PLoS ONE.

[57] Margerison-Zilko C.E., Li Y., Luo Z. (2017). Economic Conditions During Pregnancy and Adverse Birth Outcomes Among Singleton Live Births in the United States, 1990–2013. Am. J. Epidemiol..

[58] He J., Liu Z.-W., Lu Y.-P., Li T.-Y., Liang X.-J., Arck P.C., Huang S.-M., Hocher B., Chen Y.-P. (2017). A Systematic Review and Meta-Analysis of Influenza A Virus Infection During Pregnancy Associated with an Increased Risk for Stillbirth and Low Birth Weight. Kidney Blood Press. Res..

[59] Almond D., Mazumder B. (2005). The 1918 Influenza Pandemic and Subsequent Health Outcomes: An Analysis of SIPP Data. Am. Econ. Rev..

[60] Fletcher J.M. (2018). The effects of in utero exposure to the 1918 influenza pandemic on family formation. Econ. Hum. Biol..

[61] Abdoli A., Falahi S., Kenarkoohi A., Shams M., Mir H., Jahromi M.A.M. (2020). The COVID-19 pandemic, psychological stress during pregnancy, and risk of neurodevelopmental disorders in offspring: A neglected consequence. J. Psychosom. Obstet. Gynecol..

[62] Koubovec D., Geerts L., Odendaal H.J., Stein D.J., Vythilingum B. (2005). Effects of psychologic stress on fetal development and pregnancy outcome. Curr. Psychiatry Rep..

[63] DiPietro J.A., Hilton S.C., Hawkins M., Costigan K.A., Pressman E.K. (2002). Maternal stress and affect influence fetal neurobehavioral development. Dev. Psychol..

[64] Talge N.M., Neal C., Glover V. (2007). Early Stress, Translational Research and Prevention Science Network: Fetal and Neonatal Experience on Child and Adolescent Mental Health. Antenatal maternal stress and long-term effects on child neurodevelopment: How and why?. J. Child Psychol. Psychiatry.

[65] Walsh K., McCormack C.A., Webster R., Pinto A., Lee S., Feng T., Krakovsky H.S., O’Grady S.M., Tycko B., Champagne F.A. (2019). Maternal prenatal stress phenotypes associate with fetal neurodevelopment and birth outcomes. Proc. Natl. Acad. Sci. USA.

[66] Negrato C.A., Gomes M.B. (2013). Low birth weight: Causes and consequences. Diabetol. Metab. Syndr..

[67] Zhang J., Zhang Y., Huo S., Ma Y., Ke Y., Wang P., Zhao A. (2020). Emotional Eating in Pregnant Women during the COVID-19 Pandemic and Its Association with Dietary Intake and Gestational Weight Gain. Nutrients.

[68] Pak H., Süsen Y., Nazlıgül M.D., Griffiths M. (2021). The Mediating Effects of Fear of COVID-19 and Depression on the Association Between Intolerance of Uncertainty and Emotional Eating During the COVID-19 Pandemic in Turkey. Int. J. Ment. Health Addict..

[69] Bemanian M., Mæland S., Blomhoff R., Åsgeir R.K., Arnesen E.K., Skogen J.C., Fadnes L.T. (2020). Emotional Eating in Relation to Worries and Psychological Distress Amid the COVID-19 Pandemic: A Population-Based Survey on Adults in Norway. Int. J. Environ. Res. Public Health.

